# Effects of traffic light labelling and increased healthy range on beverage choices from vending machines

**DOI:** 10.1017/S1368980024000843

**Published:** 2024-04-08

**Authors:** Ryan Calabro, Eva Kemps, Ivanka Prichard, Marika Tiggemann

**Affiliations:** 1 Psychology, College of Education, Psychology and Social Work, Flinders University, Adelaide, SA 5001, Australia; 2 Health & Exercise Sciences, College of Nursing and Health Sciences, Flinders University, Adelaide, Australia

**Keywords:** Soft drink, Beverage choice, Vending machine, Traffic light system, Healthy range, Nudging

## Abstract

**Objective:**

To test whether traffic light labels and an increased range of healthy beverages, individually and in combination, can increase healthy beverage choices from vending machines.

**Design:**

Two studies (*n* 558, 420) tested whether the provision of traffic light labels (green, amber and red) and an increased range of healthy beverages (from 20 % to 50 % green options), individually and in combination, could increase healthy beverage choices from a digital vending machine display. The studies used a between-subjects experimental design, and a hypothetical beverage choice, a limitation when considering real-world applicability.

**Setting:**

Both studies utilised an online Qualtrics survey that featured a digital vending machine display.

**Participants:**

Both studies (*n* 558, 420) consisted of university students from Flinders University and individuals from a survey recruitment service.

**Results:**

Featuring traffic lights did not significantly influence beverage choices (*P* = 0·074), while increasing the healthy range (*P* = 0·003, OR = 3·27), and the combination of both, did significantly increase healthier beverage choices (*P* < 0·001, OR = 4·83).

**Conclusions:**

The results suggest that the traffic light system and increased healthy range are not maximally effective when used on their own, and benefit greatly when combined, to increase healthy beverage choices. It was suggested that the provision of traffic light labels supplied the necessary nutritional information, and the increased healthy range offered greater opportunity to act in accordance with that information. In so doing, the present findings offer a promising pathway for reducing unhealthy beverage consumption.

The consumption of soft drinks (carbonated beverages that are high in sugar) is increasing worldwide, leading to an increased prevalence of health problems related to overconsumption^(1)^, including diabetes, tooth decay and obesity^(2,3)^. Concerningly, one of the largest age groups that consume soft drinks are adolescents and young adults^(4)^, who are particularly sensitive to developing lifelong habits that can negatively impact long-term health. In response to this growing health concern, the WHO^(5)^ made a strong recommendation that people limit their daily intake of free (added) sugar to 10 % of their total energy intake, equating to roughly 50 g or 12 tsp of sugar. With soft drinks being one of the main sources of sugar in many people’s diet^(6)^, this growing health concern has highlighted the importance of reducing soft drink consumption.

Over recent years, several countries (e.g. USA, UK, Chile and NZ) have implemented strategies to reduce soft drink consumption, including taxing soft drinks^(7)^, restricting access to soft drinks^(8,9)^ and providing nutritional warning labels^(10)^. Each of these strategies has had varying levels of success. For example, while the introduction of a soft drink tax has resulted in reduced soft drink purchases in various countries, it can also shift purchasing behaviour to other high-sugar items^(11)^. In addition, while restricting access in schools can reduce soft drink consumption, it has been shown to result in increased soft drink consumption outside of school^(8,9)^. However, prominent warning labels on products high in sugar together with a tax have reduced soft drink purchases in Chile^(10)^. Collectively, current strategies mostly involve government regulation and policy change.

An alternative strategy would be to implement a change within the immediate environment in which the choice is presented. An example is the introduction of a nutritional label at the point of sale rather than on the product itself. This carries the advantage that it still offers the choice of soft drink to those who choose to consume it and does not require manufacturers to change the labels of their products.

There are a variety of nutritional labelling systems in use around the world, such as the nutrient warning, health warning, nutritional score and traffic light system. Of these, a recent review found the traffic light system to be one of the most popular and best performing at nudging consumers towards purchasing healthier food products^(12)^. This system offers an easy-to-understand visual cue using traffic light colours (red, amber and green) to denote the nutritional value of food and beverages. These colours are universally understood concepts, with green denoting ‘go’ or ‘good’, amber ‘slow down’ or ‘limit’ and red ‘stop’ or ‘avoid’. A review of the traffic light system found that it had a significant influence in promoting healthy food choices from restaurants and cafeterias^(13)^. Because of its success in these food environments, the traffic light system may also prove effective in other settings.

One environment that is a common source of soft drinks is a vending machine, which often feature a disproportionately high number of sugar-sweetened beverages^(14–16)^. Additionally, vending machines do not typically offer nutritional information at the point of purchase. Research amongst college students has found that an intervention that displays labelling information for healthy options was perceived as the most helpful, compared to price changes, or posters explaining which options were healthier^(17)^. Thus, implementing the traffic light system for beverages within a vending machine could promote healthier choices much like it has in other consumption environments. Only one study could be identified that investigates specifically the traffic light system (in isolation to other interventions such as price changes and serving size changes). Brown *et al.* ^(18)^ placed green, amber and red stickers on the shelving below snacks on five high use machines on a university campus, which resulted in a 50 % increase in green product purchases. However, the vending machine only included snack foods.

In addition to the provision of nutritional information, it is also important to consider the opportunity (or lack thereof) for someone to make a healthy choice^(19)^. This is particularly pertinent as vending machines have been found to mostly contain unhealthy options^(14–16)^. A study by Pechey *et al.* ^(20)^ investigated whether reducing unhealthy or increasing healthy options from vending machines could improve the proportion of energy purchased from healthy options. They found that decreasing the number of positions containing unhealthy drinks resulted in less energy purchased from these drinks but found inconclusive results for increasing the number of positions that contained healthy drinks. However, this study did not feature any additional nutritional information to motivate individuals to choose healthier beverages. The very few studies that have investigated both traffic light labelling in combination with increasing the range of healthy options in vending machines have shown some promising results^(21,22)^. However, no studies could be identified that offer the ability to compare the difference in impact between featuring one or the combination of the two interventions (traffic light system and increased healthy range).

Thus, the aim of the present research was to test the impact of the traffic light system, and of increasing the range of healthy beverages, and the combination of both, in a hypothetical choice experiment that utilised a digital vending machine. Study 1 tested the traffic light system on a vending machine featuring beverages representative of the range of drinks (proportion of healthy and unhealthy drinks) typically offered. Study 2 extended this by stocking the vending machine with a greater proportion of healthy options following recent guidelines from the Victorian Healthy Eating Advisory Service^(23)^ (similar to guidelines provided in the USA^(24)^). Based on previous research^(12,18)^, we predicted that the vending machine with the traffic light system would increase the percentage of healthy (green) choices in both studies. It should be noted that the beverage choice was hypothetical, and the results from the present study were intended to guide the implementation of the traffic light system on real vending machines on campus.

## Methods

### Participants

#### Study 1

Participants were 558 young adults (414 women and 143 men) with a mean age of 20·21 years (sd = 2·41). The sample consisted of 317 undergraduate students at Flinders University who took part for course credit and 241 participants from Prolific who received a small monetary reimbursement. Participation was limited to young adults (17–25 years) to capture the core consumers of soft drinks. Mean BMI of the sample was 23·56 kg/ 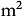 (sd = 5·20).

#### Study 2

Participants were 420 young adults (284 women and 136 men) with a mean age of 20·67 years (sd = 2·26). The sample consisted of forty-two undergraduate students at Flinders University who took part for course credit and 378 participants from Prolific who received a small monetary reimbursement. Participation was again limited to young adults (17–25 years) to capture the core consumers of soft drinks. Mean BMI of the sample was 24·30 kg/ 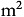 (sd = 6·40).

### Design

Both studies used a between-subjects design. For Study 1, participants were randomly allocated to one of two vending machine display conditions: traffic light system or no traffic light system (control). The dependent variable was the classification of the beverage chosen (green, amber or red). Study 2 utilised the same design, but included two additional conditions: increased healthy range and increased healthy range combined with traffic lights.

### Materials

#### Vending machine displays (Study 1)

**Fig. 1:**
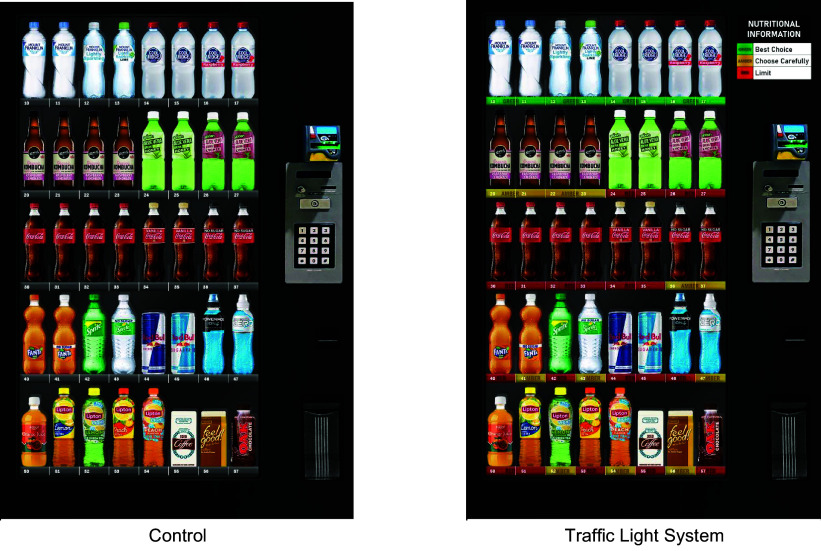
The two vending machine conditions featured in Study 1.

Two vending machine displays were created: traffic light system and control (no traffic light system). As shown in Fig. 1, the traffic light system condition featured labelling under each beverage indicating whether it was classified as either green, amber or red based on nutritional value. Text was also included in the colour classification labels to allow those who are colour blind to participate in the study. A legend in the top right-hand corner provided more information about the colour classifications, with green indicating ‘best choice’, amber ‘choose carefully’ and red ‘limit’. Drink colour classifications and the legend text for the colours were based on the Food Checker classification of the Victorian Healthy Eating Advisory Service^(23)^. This service enables beverages to be searched in an online database to find the colour classification according to set nutritional criteria. The criteria specify that green beverages are low in added sugar, energy or fat, red beverages are high in added sugar, energy or fat, and amber beverages are between the two (see Victorian Healthy Eating Advisory Service^(23)^ for more information). These criteria are similar to those included in the guidelines for healthy vending by the Centre of Disease Control and Prevention in the USA^(24)^. The control vending machine had an identical drink layout and range but did not feature the traffic light system.

The selection and placement of the drinks in the vending machine were based on Flinders University’s vending machine sales data. This resulted in eight spaces (20 %) in the vending machine layout filled with green beverages, twelve spaces (30 %) with amber beverages and twenty spaces (50 %) with red beverages. The selection of green beverages included popular varieties of water (including lightly flavoured and sparkling waters). The amber beverages included kombucha, aloe vera drinks and sugar-free soft drinks. The red beverages included soft drinks and energy drinks. All serving sizes of beverages were kept as close as possible to a 500-ml offering.

#### Vending machine displays (Study 2)

**Fig. 2:**
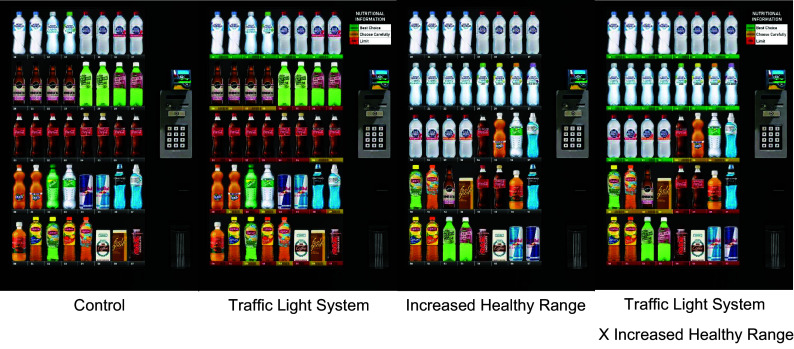
The four vending machine conditions featured in Study 2.

For the second study, we aimed to test whether an increased healthy range could promote healthier beverage choices. To design the increased healthy beverage layout for the vending machine, we turned to guidelines that have been developed for this specific purpose, of which there were several available (e.g. USA^(24)^, Canada^(25)^, UK^(26)^ and Australia^(23)^). We focused on the Australian vending machine layout guidelines^(23)^, as they were the most comprehensive and from the same source used in Study 1 to classify the beverages based on nutritional criteria. These guidelines suggest having at least 50 % green choices and no more than 20 % red choices. Therefore, the newly designed increased healthy range display featured twenty spaces (50 %) filled with green beverages, eight spaces (20 %) with amber beverages and twelve spaces (30 %) with red beverages. Compared to the range featured on the other displays, this offered a 20 % increase in the number of green choices, a 10 % decrease in the number of amber choices and a 10 % decrease in the number of red choices. To achieve these recommended percentages, the increased healthy range featured four additional green beverage choices (one additional water flavour and three additional sparkling water options) and two fewer red beverage choices (soft drinks), removed based on their very low popularity in Study 1. We also created an additional display that featured this increased healthy range in combination with the same traffic light system used in Study 1 (see Fig. 2). These additional vending machine displays enabled us to independently test the main effects of the traffic light system and an increased healthy range, as well as the combination of the two, on beverage choices.

#### Beverage choice task

In both studies, participants were asked to imagine that they were standing in front of a vending machine and to choose a beverage that they would like to drink straight away, without consideration of price or serving size. Participants made their selection by clicking on their beverage of choice. They were then asked to describe in a few words why they chose that particular beverage.

#### Background information

In both studies, participants reported their age and gender, and the last time they drank anything (estimated to the nearest 15 min). They also rated how thirsty they were on a 100-mm visual analogue scale ranging from ‘not at all thirsty’ to ‘extremely thirsty’.

#### Procedure

Both studies were conducted online using Qualtrics. After providing informed consent, participants provided background information. They then completed the beverage choice task and post-choice question. Most participants completed the study in 10–15 min.

## Results

### Study 1

#### Beverage choice

Overall participants mostly chose red beverages (57 %), followed by green beverages (23 %) and amber beverages (20 %). The most commonly reported reason for choosing a particular beverage was because it was liked (18·8 %), or because it was considered healthy (9·2 %), a favourite (8·8 %), refreshing (8·7 %) or tasty (8·7 %).

#### Effect of vending machine condition on beverage choice

From Table 1, it can be seen that there was a slight trend towards healthier beverage choices for the vending machine that featured the traffic light system, with green beverages chosen 6·1 % more frequently and red beverages chosen 6·3 % less frequently relative to the control machine. However, in both conditions most participants still chose unhealthy (red) beverages. A binary regression was conducted to test whether the traffic light condition predicted healthy (green) beverage choices, while controlling for thirst. The overall model was not significant, *X* ^2^ (2, *n* 558) = 5·347, *P* = 0·069, indicating that featuring the traffic light system did not significantly influence healthy beverage choices.

**Table 1:** Study 1 percentage of beverage colour classifications chosen in each condition

Condition	Green	Amber	Red
Control	19·6 %	20·4 %	60·0 %
Traffic light system	25·8 %	20·5 %	53·7 %

### Study 2

#### Beverage choice

As in Study 1, overall participants again mostly chose red beverages (53 %), followed by amber beverages (25 %) and green beverages (22 %). The most commonly reported reason for choosing a particular beverage was because it was liked (18·1 %), or because it was considered healthy (12·7 %), a favourite (9·9 %), tasty (8·7 %) or refreshing (7·4 %).

#### Effect of vending machine condition on beverage choice

**Fig. 3:**
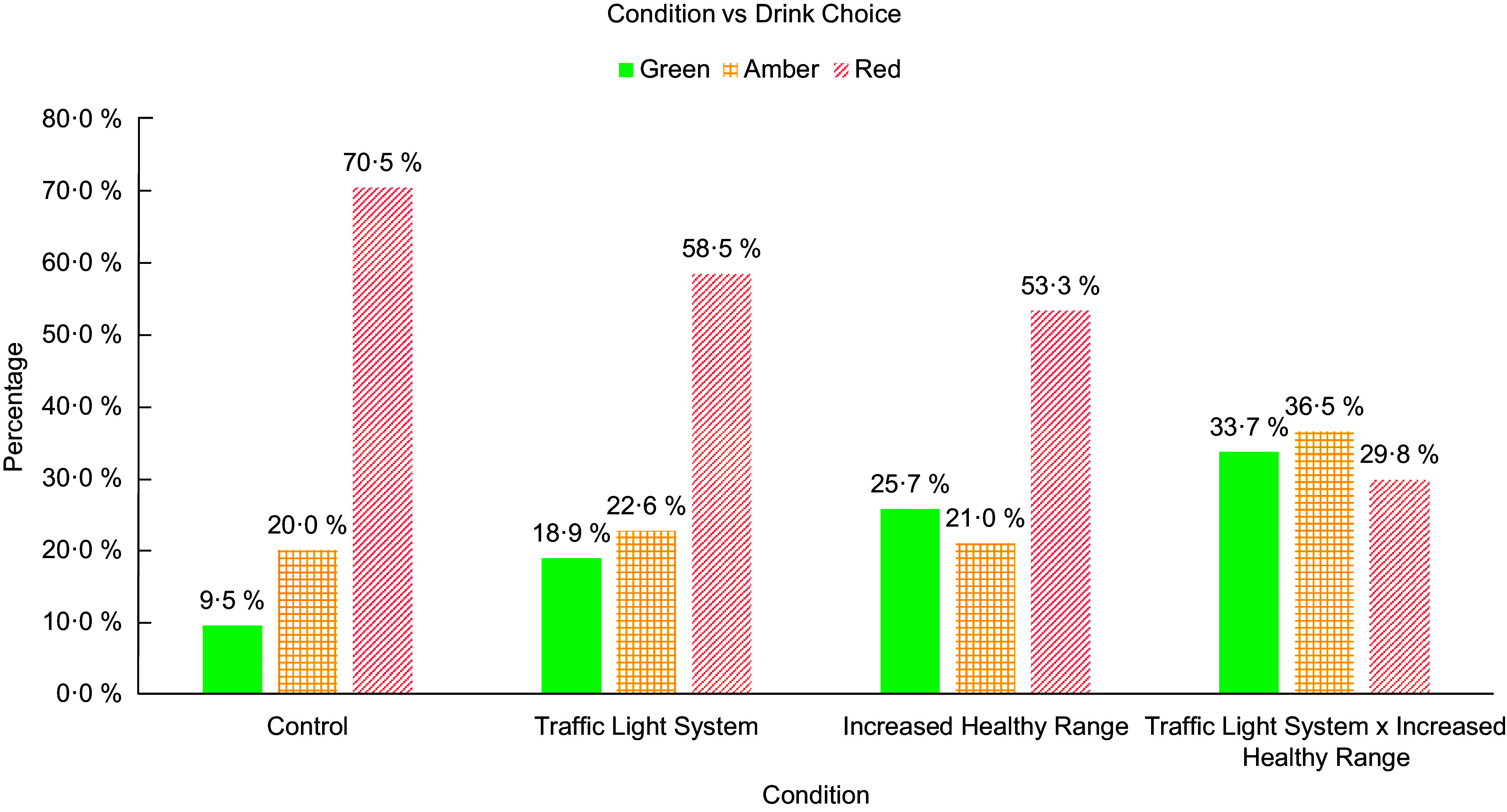
Study 2 percentage of beverage colour classifications chosen in each condition.

Figure 3 shows the beverage choices in each condition. It can be seen that red beverages were clearly the most frequently chosen in the first three conditions. In the traffic light system and increased healthy range combined condition, however, green and amber beverages were selected slightly more often than red beverages.

A binary regression was conducted to test of the effect of condition (traffic light system, increased healthy range and the combination) on the likelihood that participants chose a healthy (green) beverage while controlling for thirst. The overall model was significant, *X* ^2^ (4, *n* 420) = 25·691, *P* < 0·001. The model explained 9·1 % (Nagelkerke R2) of the variance in healthy beverage choices and correctly classified 78·1 % of cases. As can be seen in Table 2, participants in the traffic light condition were not significantly more likely to choose a healthy beverage (*P* = 0·074), while those in the increased healthy range condition were 3·2 times more likely to choose a healthy beverage, and those in the traffic light system and increased healthy range combined condition were 4·8 times more likely to choose a healthy beverage, compared to the no traffic light system and standard range (control) condition.

**Table 2:** Coefficients for the variables entered in the binary regression predicting a healthy (green) beverage choice

Predictor	se	Wald	*P*	OR	95 % CI for OR
					Lower, Upper
Traffic light system	0·417	3·192	0·074	2·106	0·930, 4·770
Increased healthy range	0·402	8·648	0·003*	3·266	1·484, 7·187
Traffic light system and increased healthy range	0·394	15·947	0·000**	4·827	2·229, 10·452
Thirst	0·006	5·163	0·023*	1·013	1·002, 1·024

* *P* < 0·05.

** *P* < 0·001.

## Discussion

The present studies examined the effects of a traffic light system and an increased healthy range, individually and in combination, on beverage choices from a vending machine. Featuring the traffic light system on the vending machine resulted in a non-significant trend towards more healthy beverage choices (Studies 1 and 2). However, increasing the range of healthy options did produce significantly more healthy beverage choices (Study 2). In addition, the combination of the traffic light system and an increased healthy range resulted in the largest proportion of healthy beverage choices.

It was predicted that featuring the traffic light system on the vending machine would result in significantly more healthy beverage choices. Although there was a trend towards healthier (green) beverages and fewer unhealthy (red) beverages being chosen when featuring the traffic light system, the effect of the traffic light system was not significant. This parallels the result of Brown *et al.* ^(18)^, although their reported increase (50 %) was much larger. These findings suggests that in settings where mostly unhealthy items are on offer (such as vending machines^(14–16)^), using traffic lights alone may not be enough to produce a substantial shift towards healthier choices. Traffic lights have been shown to be successful in increasing healthy choices in settings that typically offer a greater variety of healthy items (e.g. restaurants and cafeterias)^(13)^, increasing the likelihood of an appealing alternative. Consequently, a stronger nudge than the traffic light system may be required to produce healthier beverage choices from a vending machine environment that typically features predominantly unhealthy beverages.

In Study 2, the effect of the increased range of healthy beverages was significant and was stronger than that of the traffic light system for increasing healthy beverage choices. The range of healthy beverages offered was based on the guidelines by the Victorian Healthy Eating Advisory Service^(23)^, which mainly involved increasing the range of green beverage options and correspondingly reducing the range of ‘red’ options. The present findings show that following these or similar guidelines for the stocking of beverage vending machines could potentially lead to an increase in the percentage of healthy beverage choices and a decrease in the percentage of unhealthy beverage choices. One reason for the stronger effect compared to the traffic light system may simply be that the increased healthy range offered a greater number of appealing healthy options. It is also possible that stocking the vending machine with a majority of spaces (50 %) featuring healthy beverages may have acted as a normative cue. This result is in line with previous research that found reducing the number of positions that featured unhealthy drinks in vending machines resulted in a reduced proportion of energy purchased from these unhealthy drinks^(20)^. In the present study, although the number of spots occupied by unhealthy beverages was reduced, there was minimal reduction in the range of unhealthy options to ensure that the choice of those beverages was still available.

Importantly, the combination of featuring the traffic light system and an increased healthy range resulted in the largest increase in the percentage of healthy choices. It is possible that either alone addresses only one part of the process, whereas together they offer both easy to understand nutritional information though the traffic light system, together with sufficient opportunity to act on this information through the increased healthy range. A similar finding was reported by Kocken *et al.* ^(21)^ who found that their healthy vending machine intervention worked best when including the combination of lower calorie products and labelling (however, their lower calorie products were also lower priced). It should be noted that the combination condition here resulted in an increase in both green and amber beverage choices, the latter possibly representing people who would have originally chosen an unhealthy (red) beverage changing their choice to a healthier option (amber), for example, by choosing a sugar-free soft drink (amber) over a standard soft drink (red). A similar finding of an increase in both green and amber choices in response to labelling and stock changes in vending machines was reported by Stamos *et al.* ^(22)^. In terms of public support for interventions of this nature, there is evidence that increasing the healthy range of products in vending machines^(27)^ and featuring nutritional information^(28)^ are considered favourably. Importantly, research has shown that having healthier options in vending machines does not necessarily decrease revenue^(29–31)^. In fact, Hua *et al.* ^(29)^ note the importance of highlighting healthy options in the machine as a strategy to mitigate any potential profit loss from removing any unhealthy options.

The present studies show the importance of both increasing the healthy range and including the traffic light system to maximise the percentage of healthy beverage choices from vending machines. While increasing the healthy range offered in the vending machine resulted in a significant increase in healthy choices, this effect was less than half of that obtained by combining it with the traffic light system. Indeed, the combined condition was the only condition where the percentage of healthy (green) choices exceeded that of unhealthy (red) choices. Such an increase in the percentage of healthy beverage choices is a potentially significant step towards reducing negative health outcomes associated with the overconsumption of soft drink and other sugar-sweetened beverages. For example, on each occasion an individual chooses a sugar-free soft drink over a standard soft drink, they will consume approximately twelve teaspoons less of sugar, in turn making them more likely to meet the WHO recommendations for daily free sugar intake and avoid the health issues related to the overconsumption of soft drinks.

Although the effect of an increased healthy range in combination with traffic lights was demonstrated for vending machines, these colour coding and layout changes could also be implemented in other environments (such as drink fridges in cafes, schools and supermarket shelves) and may result in a similar increase in the percentage of healthy beverage choices. Supermarkets, in particular, have been shown to feature a disproportionately high number of unhealthy (ultra-processed) packaged foods^(32,33)^. Existing research on this intervention combination outside of vending machines is limited, with one study reporting^(34)^ that calories and macronutrient content labelling in combination with introducing lower energy-dense foods resulted in healthier eating behaviour in a workplace cafeteria. A systematic review by Schruff-Lim *et al.* ^(19)^ also highlights the need for more research investigating the combination of nutritional labels (such as the traffic light system) and an increased healthy range. The present study offers promising evidence in support of such combined interventions in the vending machine environment. Future research could test the present studies’ findings in other settings.

Like all research, the present studies have some limitations warranting acknowledgement. First, participants were presented with only a hypothetical beverage choice and never had the opportunity to consume their chosen drink. Nevertheless, hypothetical choices have been found to engage comparable brain systems as real choices^(35)^ and thus can be considered a valid predictor of actual choices. Second, it is not possible to rule out the effect of demand characteristics in the present studies. Specifically, in the conditions where beverages were labelled by the traffic light system, participants may have deduced the purpose of the research and chosen beverages accordingly. This may have magnified the effect observed compared to an intervention featured on real vending machines.

In conclusion, the present studies offer evidence supporting healthy vending machine guidelines, such as the ones available in Australia^(23)^, for stocking and providing nutritional labelling on vending machines. The resulting interventions provide a promising pathway for guiding people’s choices away from soft drinks and other unhealthy drink options towards something healthier with less or no sugar and thereby reducing sugar consumption.
